# Electroacupuncture restores intestinal mucosal barrier in IBS-D rats by modulating mast cell-derived exosomal MiR-149-5p

**DOI:** 10.3389/fimmu.2025.1641484

**Published:** 2025-09-17

**Authors:** Yujun Hou, Fangli Luo, Kai Wang, Ying Chen, Lu Wang, Yanqiu Li, Siqi Wang, Junpeng Yao, Ying Li, Siyuan Zhou

**Affiliations:** ^1^ School of Acupuncture and Tuina, Chengdu University of Traditional Chinese Medicine, Chengdu, China; ^2^ Clinical School of Integrated Traditional and Western Medicine, North Sichuan Medical College, Nanchong, China; ^3^ Chengdu First People’s Hospital, Chengdu, China; ^4^ Department of Acupuncture and Moxibustion, Hospital of Chengdu University of Traditional Chinese Medicine, Chengdu, China

**Keywords:** irritable bowel syndrome, electroacupuncture, mast cells, exosomes, micrornas, tight junctions

## Abstract

**Background:**

Intestinal barrier dysfunction is a key etiology of diarrhea-predominant irritable bowel syndrome (IBS-D), and our previous work has demonstrated that mast cells play a critical role in this process. Here, we further show that electroacupuncture (EA) restores intestinal mucosal barrier in IBS-D Rats by modulating mast cell-Derived exosomal (MC-EXO) microRNAs (miRNAs).

**Methods:**

IBS-D was induced in rats using chronic unpredictable mild stress (CUMS) combined with Senna solution administration, and confirmed through assessments of visceral pain threshold, diarrhea index, percentage of time spent in open arms, hematoxylin and eosin staining was performed to evaluate the pathological features of the colon. Model rats were treated with EA in combination with the mast cell agonist C48/80, CRF-R1 agonist Ucn1, or exosome antagonist GW4869. CRF and CRF-R1 mRNA expression levels were measured using qPCR, and mast cell activation and degranulation were examined by transmission electron microscopy (TEM) and immunohistochemistry (IHC). Additionally, intestinal barrier integrity and tight junction expression were evaluated by ELISA, TEM, Western blot (WB), and IHC. MC-EXO miRNAs were extracted, sequenced, and subjected to Gene Ontology (GO) and Kyoto Encyclopedia of Genes and Genomes (KEGG) enrichment analyses. Furthermore, Caco-2 cells were transfected with miR-149-5p and miR-22-5p mimics to determine the effect of these miRNAs on intestinal permeability and tight junction protein expression. To further validate the effect of miR-149-5p, IBS-D rats were administered adeno-associated viruses (AAV) overexpressing miR-149-5p, and mast cell activation and intestinal barrier function were evaluated.

**Results:**

EA alleviated IBS-D symptoms by downregulating CRF and CRF-R1 expression, inhibiting mast cell activation, and upregulating tight junction protein expression. These effects were abrogated by CRF and mast cell agonists, but enhanced by an exosome inhibitor. MiRNA sequencing revealed significantly higher miR-149-5p and miR-22-5p expression levels in the model group compared to the EA group. KEGG and GO enrichment analyses showed that these miRNAs were enriched in pathways associated with tight junctions. Transfection of Caco-2 cells with miR-149-5p or miR-22-5p mimics increased monolayer permeability and downregulated the expression of tight junction proteins. Additionally, administration of AAV-miR-149-5p abolished the protective effect of EA in IBS-D rats.

**Conclusion:**

MC-EXO miR-149-5p modulates EA-mediated intestinal barrier repair in IBS-D rats.

## 1 Background

Irritable bowel syndrome (IBS) is a prevalent functional gastrointestinal (GI) disorder that affects millions of individuals worldwide (1), imposing significant medical and economic burdens (2). IBS is classified into subtypes based on stool consistency, with diarrhea-predominant IBS (IBS-D) being one of the most common forms (3). While dietary modifications, lifestyle changes, and pharmacologic treatments have been recommended as first-line treatments for IBS-D, their overall efficacy remains suboptimal (4). Therefore, a deeper understanding of the pathogenesis of IBS-D, particularly the roles of the intestinal barrier and immune response, is crucial for advancing therapeutic strategies and improving patient outcomes.

Normal immune function is essential for the maintenance of intestinal barrier integrity (5). There is growing evidence suggesting that mast cells, a key component of the innate immune system, contribute to intestinal barrier homeostasis (6). Notably, mast cells have been shown to play important roles in the pathogenesis of IBS-D. Mucosal mast cells are aberrantly activated in patients with IBS-D, and the extent of their activation correlates with both intestinal permeability and the severity of IBS-D symptoms (7). Degranulation is the primary mode of mast cell activation, triggering inflammatory responses and barrier damage (8). The mediators released during this process can degrade tight junction proteins, leading to disruption of the intestinal barrier (9). Corticotropin-releasing factor (CRF), a hormone produced by the hypothalamic paraventricular nucleus, is a key regulator of the stress response and mast cell activation via the CRF receptor 1 (CRF-R1) (10, 11). Studies have shown that CRF, CRF-R1, and mast cells are involved in the regulation of intestinal barrier functions in IBS-D (12–14).

Acupuncture is a major component of traditional Chinese medicine (TCM) that has been proposed as a potential treatment for IBS-D (15). Previous studies from this laboratory revealed that electroacupuncture (EA) attenuated IBS-D by downregulating CRF-R1 expression in both hypothalamic and colonic tissues, alleviating anxiety- and depression-like behaviors, upregulating the expression of tight junction proteins, and inhibiting mucosal mast cell hyperactivation (16, 17). However, the exact mechanisms by which mast cells and their downstream targets drive disease development warrant further investigation (18).

Exosomes are microvesicles secreted by cells that contain a wide array of microRNAs (miRNAs) and other bioactive substances, reflecting the cellular state (19) and facilitating intercellular communication (20, 21). In patients with IBS, mast cell-derived exosomes (MC-EXOs) have been reported to be positively correlated with symptom severity (22), suggesting that MC-EXOs may participate in IBS pathogenesis. *In vitro* experiments have demonstrated that MC-EXOs downregulate Claudin-8 protein expression and increase intestinal permeability by transferring miR-223 to intestinal epithelial cells (IECs) (23). These findings suggest that MC-EXOs and their miRNA cargo may represent potential therapeutic targets for IBS.

The present study evaluated the role of MC-EXOs miRNAs in EA-mediated repair of intestinal barrier in a rat model of IBS-D. It was hypothesized that EA may exert its therapeutic effects by modulating MC-EXO miRNA expression, thereby enhancing tight junction protein expression, restoring intestinal barrier integrity, and ultimately alleviating IBS-D symptoms.

## 2 Materials and methods

### 2.1 Animals

Forty-eight female Sprague Dawley rats (8 weeks, 240 ± 10 g) were purchased from Chengdu Dashuo Biotech Co. Ltd. (Chengdu, China) and housed in the SPF facility of the Experimental Animal Center of Chengdu University of Traditional Chinese Medicine. The animals were maintained under controlled humidity, temperature, and a regulated circadian rhythm. The experimental protocols were approved by the Experimental Animal Welfare Ethics Committee at the Chengdu University of Traditional Chinese Medicine (ref. no.2021-15).

### 2.2 IBS-D induction and treatment

**Figure 1:**
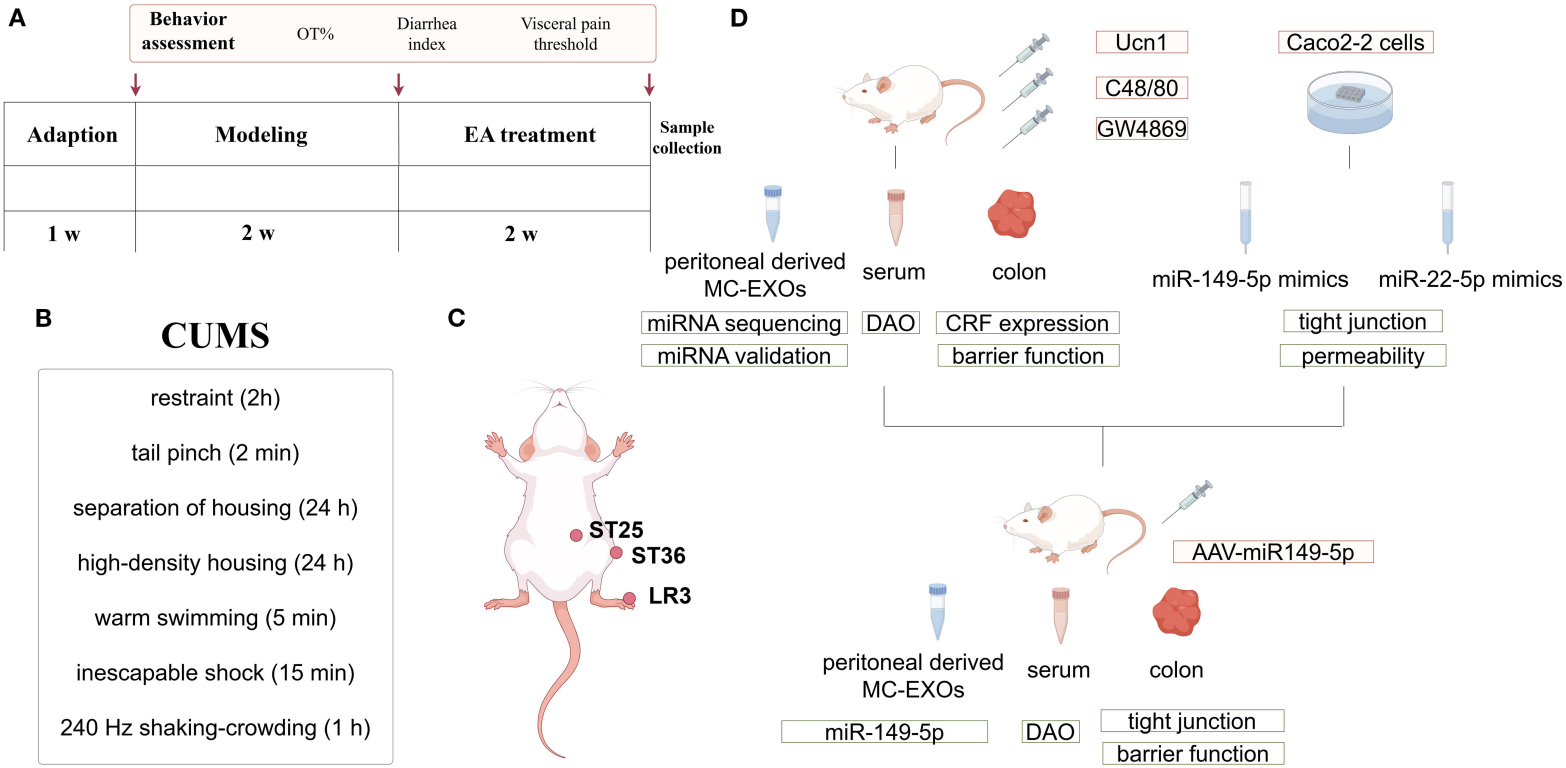
Schematic diagram of the experimental protocol: **(A)** Flow diagram of the *in vivo* experimental procedure. **(B)** The CUMS procedure. **(C)** Location of acupoints for EA treatment. **(D)** Experimental design. Ucn1, Urocortin 1, a CRF1 receptor agonist; C48/80, Compound 48/80, a mast cell agonist; GW4869: an exosome antagonist; MC-EXOs, mast cell-derived exosomes.

After one week of acclimation, rats were randomly divided into the control group, model group, EA group, EA + C48/80 group (mast cell agonist), EA + Ucn 1 group (CRF agonist), and EA + GW4869 group (exosome antagonist), with 8 rats per group. Except for the control group, IBS-D was induced in all other groups by administering 0.3 g/mL of Senna solution via oral gavage at a dose of 10 mL/kg, combined with chronic unpredictable mild stress (CUMS), for 14 days (24) (**Figure 1B**). EA treatment was performed once daily for 14 days in the EA, EA+Ucn1, EA+C48/80, and EA+GW4869 groups, whereas rats in the control and model groups were restrained using the same apparatus without receiving EA. At 30 minutes prior to each EA treatment, rats in the respective groups were administered Ucn1 (Sigma, tail vein injection, 10 μg/kg), C48/80 (Sigma, intraperitoneal injection, 0.75 mg/kg) or GW4869 (MCE, intraperitoneal injection, 1.25 mL/kg) (**Figure 1D**).

### 2.3 EA intervention

EA was performed at ST36, ST25, and LR3 as previously described (24). ST36 is located on the lateral aspect of the knee, approximately 5 mm below the fibula head. ST25 is situated 5 mm lateral to the umbilicus. LR3 is found on the dorsum of the foot, between the first and second metatarsal bones (**Figure 1C**). After rats were restrained and the skin was disinfected, stainless steel acupuncture needles (Hwato, Suzhou Medical Supplies Co., Ltd., Φ0.13 × 13 mm) were inserted into the selected acupoints at a depth of 1–2 mm. The needles at ST25 and ST26 were connected to an EA apparatus (HANS-200A, Nanjing, China), with stimulation delivered using alternating sparse and dense waves at an intensity of 1.5 mA and a frequency of 2 Hz/15 Hz. EA was performed daily on alternating hind limbs, with each session lasting 20 min (**Figure 1A**). Out of the three waveforms, disperse-dense waves were the least tolerated but frequently utilized; Moreover, the pain-relieving effectiveness at a frequency of 2 Hz/15 Hz surpasses that of 2 Hz/100 Hz. Our earlier research demonstrated that choosing these acupoints and using specific EA parameters can alleviate IBS-D symptoms.

### 2.4 Behavioral assessments

Behavioral assessments were conducted before IBS-D induction, after IBS-D induction, and after EA treatment. Research has shown that patients with IBS have increased risk of anxiety and depression (25). Visceral hypersensitivity, diarrhea, and anxiety-like behaviors were evaluated using the visceral pain threshold, diarrhea index, and percentage of time spent in open arms (OT%), respectively, as previously described (24).

### 2.5 Sample collection

Rats were euthanized at the end of treatment, and the distant colon (5 cm to the anus), serum, and peritoneal wash (using 10 mL D-Hank’s solution) were collected for various assays (**Figure 1D**).

### 2.6 Histology

Pathological changes in the colon tissues were examined by H&E staining. Briefly, colon tissues were fixed in 4% paraformaldehyde, dehydrated in an automatic dehydrator, embedded in paraffin, sectioned, stained with H&E, mounted, and examined using the Pannoramic 250 digital slide scanner (3DHISTECH, Hungary) at 100× magnification.

### 2.7 Enzyme-linked immunosorbent assay

Serum levels of diamine oxidase (DAO), a sensitive marker for intestinal barrier function, were quantified using an ELISA kit per the manufacturer’s instructions (Rat DAO ELISA KIT, ZCI BIO, China). After adding samples, washing, color developing and adding terminating solution, OD values were measured with a microplate reader.

### 2.8 Western blot

The colonic expression levels of the tight junction proteins ZO-1, Occludin, and Claudin-1 were measured using WB. Briefly, total protein was extracted from colonic tissues using RIPA lysis solution (Servicebio, China) and quantified by the BCA protein quantification kit (Beyotime, China). Equal quantity of protein samples were loaded for SDS-PAGE, and the separated proteins were transferred onto a polyvinylidene difluoride (PVDF) membrane (Sigma-Aldrich, USA). The PVDF membrane was blocked with 5% skimmed milk, washed, incubated with anti-ZO-1, anti-Occludin (1:1000, 1:1000, Abcam, UK), anti-Claudin-1, and anti-β-actin antibodies (1:2000, 1:50000, Abclonal, China) at 4°C overnight; after washed with TBST, the PVDF membrane was incubated with biotinylated goat anti-rabbit IgG (H + L) secondary antibody (1:5000; Abclonal, China), washed, and incubated with chromogenic substrate solution at room temperature for 2~3 h. The protein bands were visualized using Tianeng GIS chassis control software V2.0 (China), and the relative expression levels of ZO-1, Occludin, and Claudin-1 were quantified by normalization to β-actin.

### 2.9 Immunohistochemistry

Immunohistochemistry (IHC) was performed to assess the expression of colonic tight junction proteins and tryptase, a marker of mast cell activation. Colon tissues were immersion-fixed in 4% paraformaldehyde (Sinopharm Chemical Reagent, China) for 72 h, embedded in paraffin, cut into 5-µm-thick sections, incubated with 3% hydrogen peroxide solution (Sinopharm Chemical Reagent, China) for 10 min, and blocked with 10% goat serum (Beijing Zhongshan Golden Bridge Biotechnology, China) for 2 h at 37°C. Primary antibodies (Occludin, 1:100, Abcam, UK; ZO-1, 1:200, Servicebio, China) were added dropwise and incubated overnight at 4°C; after washed with PBS, A secondary antibody (HRP-labeled goat anti-rabbit, 1:100, Servicebio, China) working solution was added dropwise and incubated at 37 °C for 30 min. DAB substrate kit (Beijing Zhongshan Golden Bridge Biotechnology, China) was used for the color-reaction and nuclei counterstain was performed with hematoxylin (J&K Scientific, China). The percentage of positive staining in each image was calculated using Halo data analysis system.

### 2.10 Transmission electron microscopy

The ultrastructure of mast cells in the intestinal mucosa was examined using TEM. Colon tissues were fixed in 2.5% glutaraldehyde, dehydrated, infiltrated, and embedded in resin. Ultrathin sections were then prepared, stained with lead citrate, and examined under a transmission electron microscope.

### 2.11 Quantitative real-time PCR

The mRNA expression levels of CRF and CRF-R1 in the colon were measured by qPCR. Total RNA was extracted from colon tissues, assessed for purity, and reverse transcribed into cDNA for PCR amplification. The primer sequences are detailed in **Table 1**.

**Table 1:** CRF and CRF-R1 primer sequences.

Primer name	Forward	Reverse
β-actin	GGGAAATCGTGCGTGACATT	GCGGCAGTGGCCATCTC
CRF	CCAGCAACCTCAGCCGATTCTG	GAGCAGCGGGACTTCTGTTGAG
CRF-R1	AGCCCGTGTGAATTATTCTGAGTGC	GCAGTGACCCAGGTAGTTGAGATG

### 2.12 Isolation and identification of peritoneal mast cells

Peritoneal fluid was collected by lavage with 10 mL D-Hank’s solution, and mast cells were isolated using Percoll density gradient centrifugation. To prepare the Percoll separation solution, Percoll was first mixed with 8.5% NaCl solution at a 9:1 ratio to achieve physiological osmotic pressure (100% stock solution), then diluted with 0.85% NaCl solution to generate the 75% and 70% Percoll separation solutions. Peritoneal wash samples were centrifuged at 1200 r/min for 5 min, and the pellets were resuspended in 1 mL PBS, following by the addition of the Percoll separation solution. Samples were then centrifuged at 1790 r/min for 20 min, and the cell layer at the interface between PBS and the Percoll separation solution was carefully transferred to a clean 1.5mL EP tube. The collected cells were centrifuged again at 1400 r/min for 5 min, treated with red blood cell lysis buffer at room temperature for 3 min, and centrifuged once more at 1400 r/min for 5 min. The cells were washed with ice-cold washing buffer and PBS, and then stained with toluidine blue for mast cell identification.

### 2.13 Extraction and purification of peritoneal MC-EXOs

The cell culture supernatant of isolated mast cells was collected, centrifuged to remove debris and was thawed rapidly in a 37°C water bath, transferred to a new centrifuge tube, and centrifuged at 2,000g for 30 min. The supernatant was carefully transferred to a new centrifuge tube and centrifuged at 10,000g for 45 min to remove larger vesicles. The supernatant was collected and was filtered through membrane filter (0.45 µm), the filtrate was centrifuged at 100,000g for 70 min, after supernatant removal and resuspended in 10 mL precooled PBS, the precipitated complex was centrifuged at 100,000g for 70 min. A total of 20µL of the final cell suspension was used for TEM, 20µL for particle size analysis, and 20µL for fluorescence-based detection of exosome-FLAG protein expression. The remaining samples were stored at -80°C for subsequent analyses.

### 2.14 High-throughput sequencing and enrichment analyses of peritoneal MC-EXOs

Total RNA was extracted from peritoneal MC-EXOs for library construction and sequenced using the Illumina Hiseq Xten platform. Total RNA samples were quality-checked using RNA Pico Chips on an Agilent 2100 bioanalyzer (Agilent technologies, US). Sequencing was performed according to the Illumina Xten User Guide Manual, the process was controlled by the data collection software provided by Illumina, and real-time data analysis was performed. Differentially expressed miRNAs (DE-miRNAs) were identified based on a threshold of P ≤ 0.05 and fold change ≥ 2. GO and KEGG enrichment analyses were subsequently performed on the identified DE-miRNAs.

### 2.15 qPCR validation of DE-miRNAs

The expression levels of miR-22-5p, miR-1-3p and miR-149-5p in the extracted MC-EXOs were validated using qPCR. The primer sequences are listed in **Table 2**.

**Table 2:** Primer sequences for DE-miRNAs.

Primer name	Sequence
U6-F	CTCGCTTCGGCAGCACA
U6-R	AACGCTTCACGAATTTGCGT
miR-22-5p-F	AGTTCTTCAGTGGCAA
miR-1-3p-F	TCGGCAGGTGGAATGTAAAGAAGT
miR-149-5p-F	TCTGGCTCCGTGTCTTC
common downstream primer for miR	CAGTGCAGGGTCCGAGGTAT

### 2.16 Administration of miR-149-5p-overexpressing adeno-associated viruses

Adeno-associated virus (AAV) vectors overexpressing miR-149-5p were constructed by Hanheng Biological Co. Ltd (Shanghai, China). The virus titer was 1012 vg/mL, and the stock was stored at -80°C. Prior to IBS-D induction, rats in the EA+AAV-miR group and EA+AAV-NC group received intraperitoneal injections of 200 μL of AAV-miR-149-5p and control AAVs, respectively. Following AAV administration, the rats in both groups underwent the same experimental procedures described in **Figure 1A**.

### 2.17 *In vitro* experiments

Caco-2 cells were cultured until a dense monolayer was formed in a transwell, then treated for 24 h at 37°C and 5% CO_2_ with either vehicle control, miR-22-5p mimics, or miR-149-5p mimics. After treatment, the medium was replaced with fresh maturation medium, and the cells were cultured for another 24 h under the same conditions to complete the transfection process. MiRNA and tight junction protein expression levels were measured using qPCR and WB, respectively. Apical delivery preserved monolayer integrity critical for functional assays.

Monolayer permeability was assessed using fluorescein isothiocyanate-dextran 40 (FD-40). Briefly, the culture medium was removed from the upper and lower chambers, and 500µL of Hank’s solution containing 50µg of FD-40 was added to the upper chamber. After two hours of incubation, the solution from the lower chamber was collected, and fluorescence intensity was measured using a microplate reader to calculate FD-40 concentration. The full experimental workflow is illustrated in **Figure 1D**.

### 2.18 Statistical analysis

Statistical analyses were conducted using SPSS 26.0 and Graphpad Prism 8.0.2. For normally distributed data with homogenous variance, one-way analysis of variance (One-way ANOVA) with FDR correction was used for multi-group comparisons to compare differences among groups, *P*<0.05 defined significance. For normally distributed data with heterogenous variance, the Kruskal-Wallis rank‐sum test was applied. *Post-hoc* pairwise comparisons were performed using the least significant difference (LSD) method. Data are presented as mean ± standard deviation (SD).

## 3 Results

### 3.1 EA alleviates IBS-D symptoms and restores intestinal function

**Figure 2:**
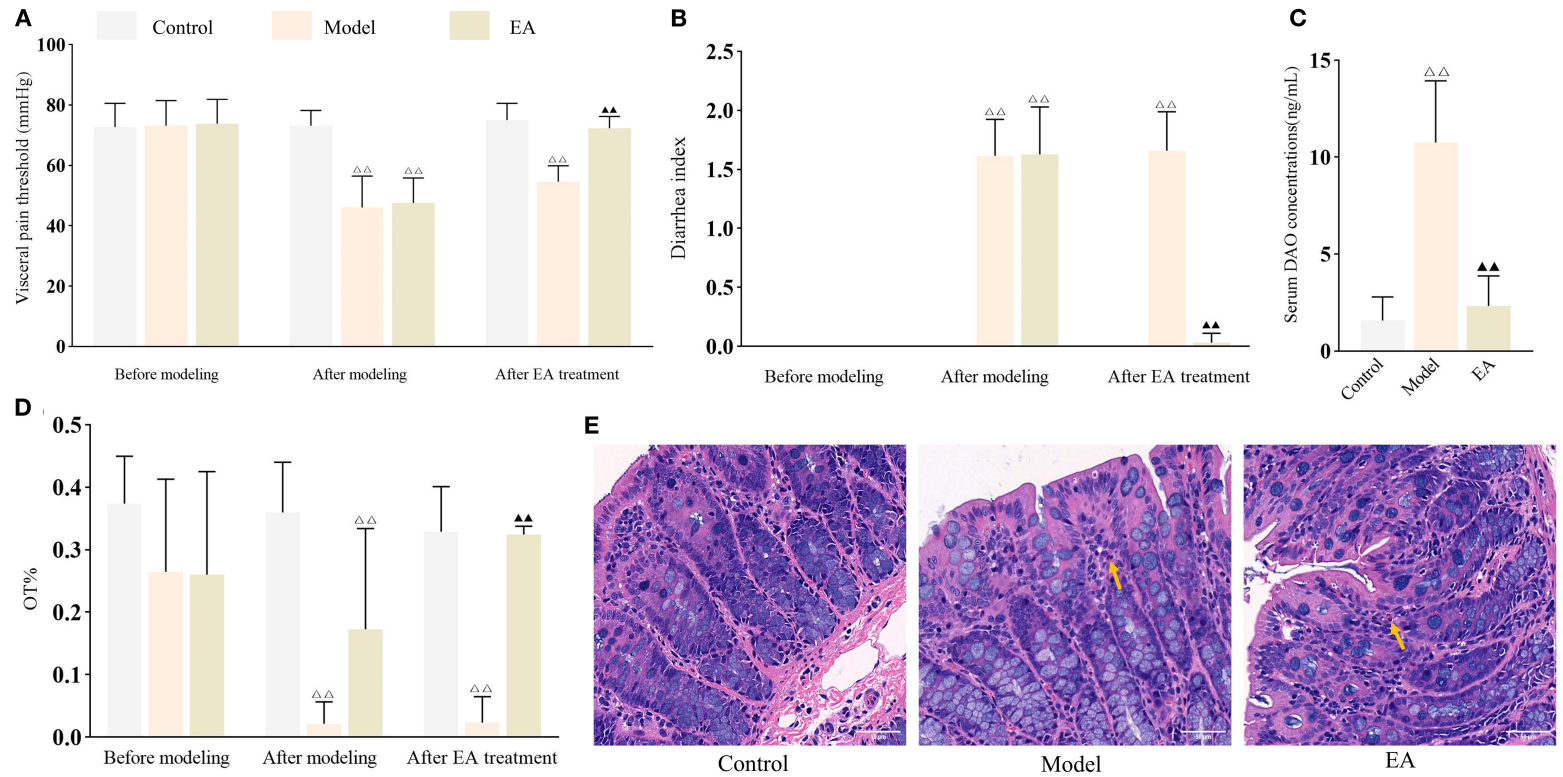
EA alleviates IBS-D symptoms and reduces intestinal permeability. Bar: mean ± SE, ^△△^ *P* < 0.01 vs. control group, ^▲▲^ *P* < 0.01 vs. model group. **(A)** Visceral pain threshold in each group at three time points, n = 8; **(B)** DI in each group at three time points, n = 8; **(C)** Serum DAO concentrations in each group, n = 6; **(D)** OT% in each group at three time points, n = 8; **(E)** Histology images of colon tissues in each group (400×). Yellow arrow indicates presence of neutrophils. Scale bar = 50 µm.

Visceral hypersensitivity, a key feature that distinguishes IBS from other gastrointestinal diseases, is commonly measured by the visceral pain threshold. Additionally, diarrhea is a hallmark symptom that differentiates IBS-D from other IBS subtypes, and its severity is evaluated using the diarrhea index (DI). OT%, a commonly used indicator of mood disorders, has been shown to be inversely correlated with the degree of anxiety. As shown in **Figures 2A, B, D**, visceral pain threshold, DI and OT% were comparable across groups before IBS-D induction (all *P* > 0.05). However, following disease induction, both the model and EA groups exhibited significantly lower pain threshold and OT% (both *P* < 0.01) and higher DI (*P* < 0.01) compared to the control group. After EA treatment, pain threshold and OT% (both *P* < 0.01) were significantly increased, while DI was decreased (*P* < 0.01), compared to untreated model rats.

As a functional gastrointestinal disorder, IBS does not typically cause structural abnormalities in the colon. Consistent with this, histological analysis revealed no marked pathological changes in the colonic tissues in any group, with occasional scattered neutrophils observed in the interstitial space of the model and EA groups (**Figure 2E**). The colonic architecture in each group remained well-preserved, with clearly defined layers and intact epithelium, consistent with the characteristics of a functional gastrointestinal disorder.

Serum DAO is a sensitive marker of intestinal permeability. As shown in **Figure 2C**, serum DAO concentrations were significantly elevated (*P* < 0.01) in the model group compared to the control group, but markedly reduced (*P* < 0.01) in the EA group compared to the model group, demonstrating that EA effectively promoted restoration of intestinal barrier integrity.

### 3.2 EA improved intestinal barrier function in IBS-D rats by inhibiting mast cell activation and exosome secretion and reducing intestinal permeability

**Figure 3:**
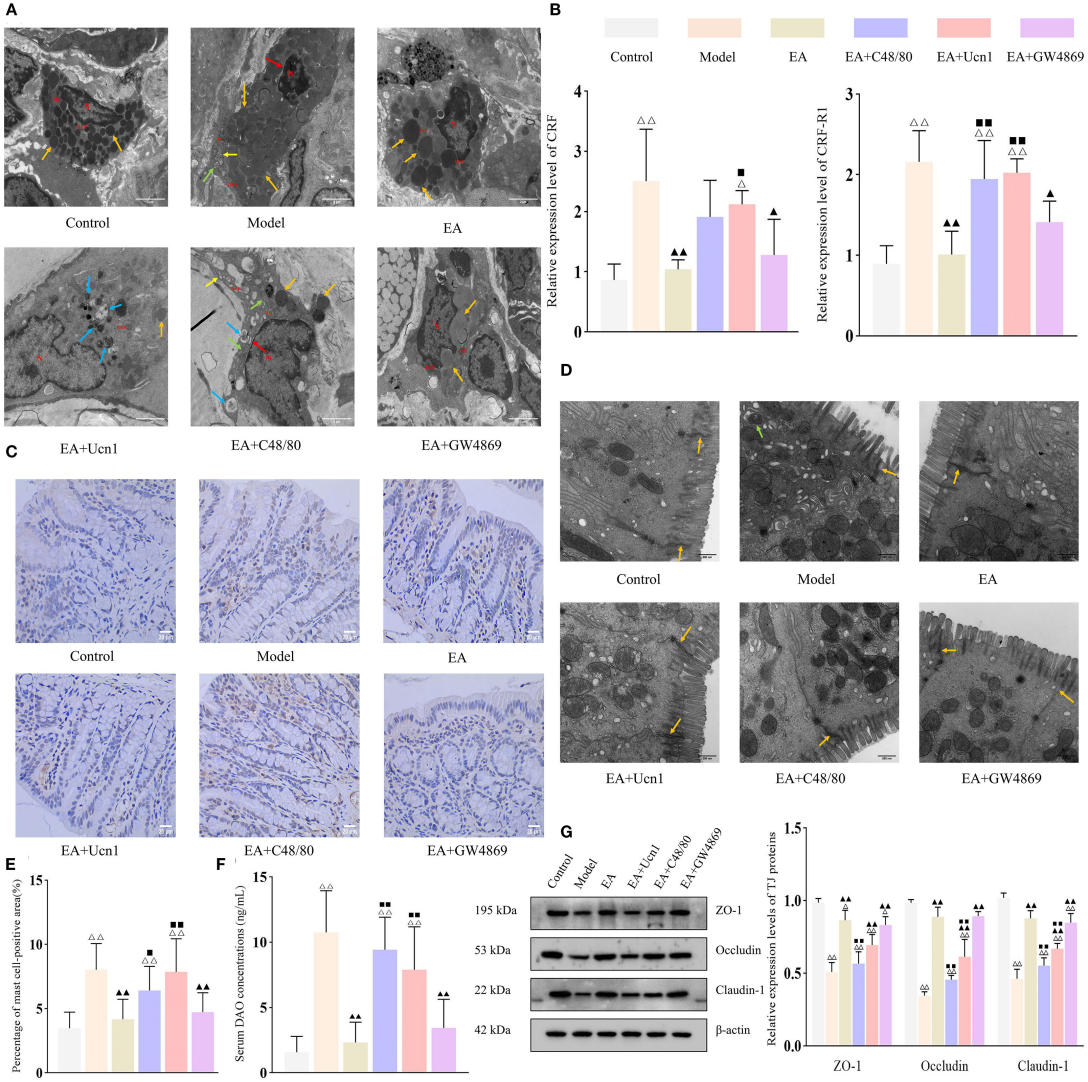
EA enhanced intestinal barrier function by downregulating CRF/CRF-R1 expression and inhibiting mast cell activation. Bar: mean ± SE, ^△△^ *P* < 0.01, ^△^ *P* < 0.05 vs. the control group, ^▲▲^ *P* < 0.01, ^▲^ *P* < 0.05 vs. the model group, ^◼◼^ *P* < 0.01, ^◼^ *P* < 0.05 vs. the EA group. **(A)** Ultrastructural of mast cells under TEM (12,000×), N, nucleus; Mi, mitochondrion; RER, rough endoplasmic reticulum. Orange arrow: secretory granules, yellow arrow: endoplasmic reticulum, green arrow: mitochondrial swelling, blue arrow: mild autophagy, red arrow: widened perinuclear space, scale bar = 2 µm; **(B)** Relative expression of CRF and CRF-R1 in each group, n = 5; **(C)** Expression of tryptase in the colon tissue (400×), scale bar = 20 µm; **(D)** Ultrastructural of tight junctions under TEM (30,000×). Orange arrow: tight junctions, green arrow: mild autophagy, scale bar = 500 nm; **(E)** Percentage of mast cell-positive area, n = 5; **(F)** Serum DAO concentrations in each group, n = 6; **(G)** Expression levels of tight junction proteins in each group, n = 5.

Previous work from this laboratory demonstrated the importance of CRF signaling and mast cell activation in EA-mediated restoration of intestinal barrier functions in IBS-D rats. qPCR analysis showed that CRF and CRF-R1 expression levels were significantly upregulated in the model group compared to the control group (both *P* < 0.01), but markedly downregulated in the EA and EA+GW4869 groups relative to the model group (both *P <* 0.01) (**Figure 3B**). Although CRF and CRF-R1 expression levels were also decreased in the in EA+Ucn1 and EA+C48/80 groups, the differences were not statistically significant (*P* > 0.05). Moreover, CRF and CRF-R1 expression levels were significantly higher in the EA+Ucn1 and EA+C48/80 groups (*P* < 0.01), but remained unchanged in the EA+GW4869 group (*P* > 0.05), compared to the EA group.

TEM was performed to examined the ultrastructural of mucosal mast cells. Mast cells in the control and EA groups displayed normal morphology and intact cellular structures. In contrast, the model group exhibited secretory granules, mild mitochondrial swelling, and a widened perinuclear space. The EA+Ucn1 group showed pronounced degranulation and autophagy activation, whereas the EA+C48/80 group had widened perinuclear space, mild autophagy, mitochondrial swelling, and dilated endoplasmic reticulum. The EA+GW4869 group exhibited only mild signs of autophagy (**Figure 3A**).

To further evaluate mast cell activation, tryptase expression was quantified by IHC (**Figure 3C**). Compared to the control group, the percentage of mast cell-positive area was significantly increased in the model group (*P* < 0.01); compared to model group, it markedly decreased in the EA and EA+GW4869 groups (both *P* < 0.01), and unchanged in the EA+C48/80 and EA+GW4869 groups (both *P* > 0.05). Compared to the EA group, the percentage of mast cell-positive area was significantly higher in the EA+Ucn1 and EA+C48/80 groups (both *P* < 0.01), and unchanged in the EA+GW4869 group (**Figure 3E**).

TEM of the intestinal epithelium revealed orderly arranged epithelium cells with intact tight junctions in the control group. On the other hand, epithelial cell apoptosis, mild autophagy, and disrupted tight junctions were observed in the model group. Tight junctions remained intact in the EA and EA+GW4869 groups but were compromised in the EA+Ucn1 and EA+C48/80 groups (**Figure 3D**). Consistent with these observations, the relative expression levels of ZO-1, Occludin, and Claudin-1 were significantly lower in the model group than in the control group (*P* < 0.01), but higher in the EA and EA+GW4869 groups (both *P* < 0.01) and similar in the EA+Ucn1 and EA+C48/80 groups (both *P* > 0.05) relative to the model group. Compared to the EA group, tight junction protein expression was significantly decreased in the EA+Ucn1 and EA+C48/80 groups (both *P* < 0.01) and comparable in the EA+GW4869 group (*P* > 0.05) (**Figure 3G**).

Similarly, serum DAO concentrations were markedly elevated in the model group relative to the control group (*P* < 0.01), but significantly decreased in the EA and EA+GW4869 groups compared to the model group. Additionally, EA+Ucn1 and EA+ C48/80 groups exhibited significantly higher serum DAO concentrations relative to the EA group (*P* < 0.01) (**Figure 3F**).

### 3.3 EA modulated miRNA expression in peritoneal MC-EXOs

**Figure 4:**
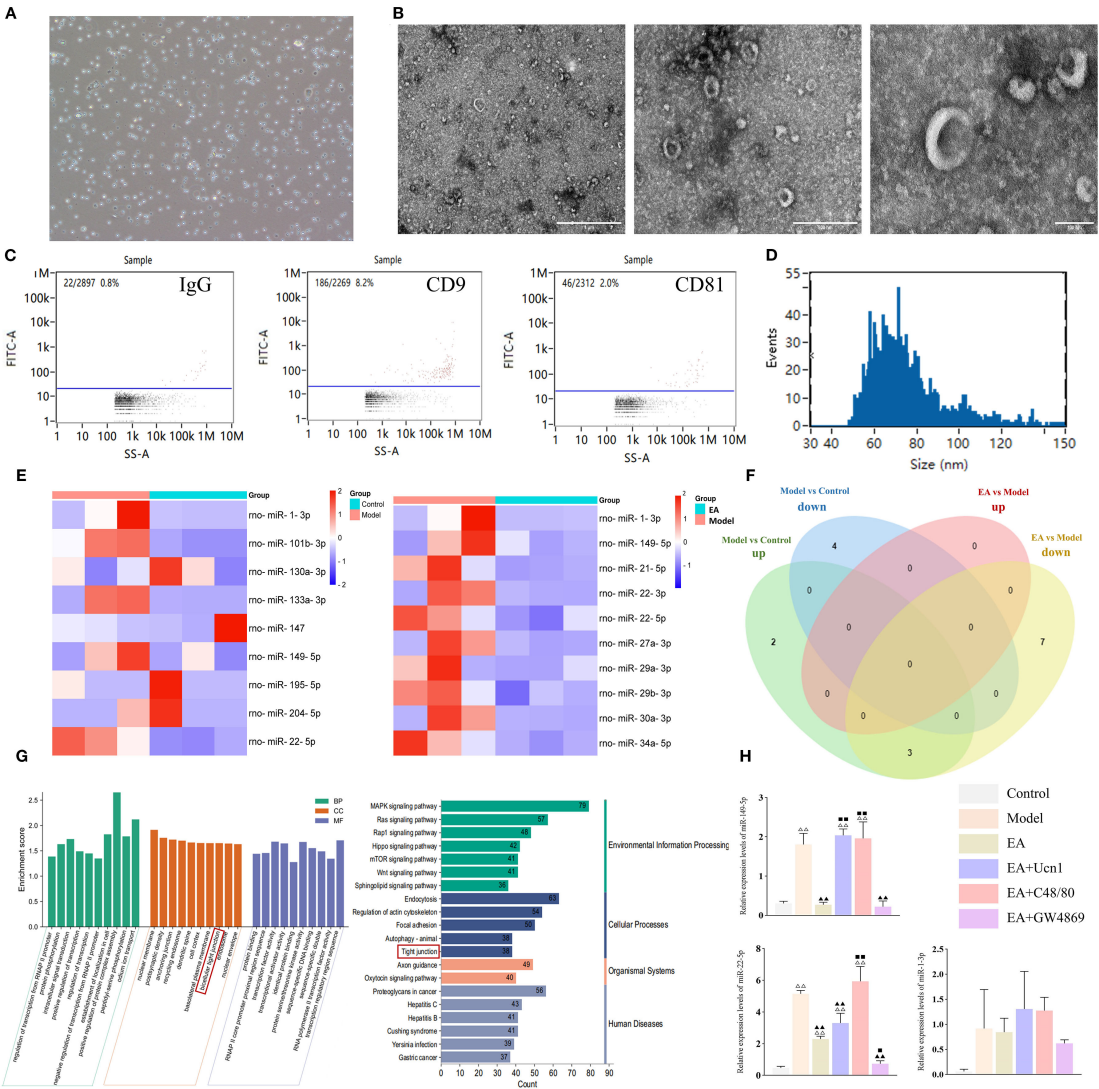
EA modulated miRNA expression levels in MC-EXOs. **(A)** Microscopic image of mast cells in culture (100×); **(B)** Ultrastructural of MC-EXOs under TEM, scale bar = 1 µm (left), 500 nm (center), 100 nm (right). **(C)** Flow plots of IgG (negative control), CD9 and CD81 expression on MC-EXOs; **(D)** Nano-flow cytometry analysis of MC-EXO particle size; **(E)** Heatmaps of DE-miRNAs between the control and model groups, and between the model and EA groups; **(F)** Venn diagram of DE-miRNAs in the control, model, and EA groups; **(G)** GO and KEGG enrichment analyses of DE-miRNAs, BP, biological processes; CC, cell components; MF, molecular functions; **(H)** Relative expression of DE-miRNAs in each group measured by qPCR. Bar: mean ± SE, n = 5, ^△△^ *P* < 0.01, ^△^ *P* < 0.05 vs. the control group, ^▲▲^ *P* < 0.01 vs. the model group. ^◼◼^ *P* < 0.01, ^◼^ *P* < 0.05 vs. the EA group.

Rat peritoneal mast cells were isolated and cultured *ex vivo*. The cells displayed a characteristic round or oval morphology, grew in clusters, and stained positively with toluidine blue, confirming their identity as mast cells (**Figure 4A**). TEM examination of MC-EXOs revealed disk-shaped vesicles of varying sizes with a central concavity (**Figure 4B**). Nano-flow cytometer showed that the MC-EXOs had a mean diameter of 75.33 (30 to 150) nm, with a particle concentration of 2.94 × 10^9^ particles/mL (**Figure 4D**). Furthermore, the MC-EXOs were positive for the exosomal markers CD9 and CD81 (**Figure 4C**), confirming successful isolation of peritoneal MC-EXOs.

Next, high-throughput sequencing was performed to identify differentially expressed miRNAs (DE-miRNAs) in MC-EXOs across the experimental groups. Compared to the control group, the model group exhibited five upregulated and four downregulated miRNAs. Additionally, ten miRNAs were significantly upregulated in the model group compared to the EA group (**Figure 4E**). Venn diagram analysis revealed that miR-22-5p, miR-149-5p, and miR-1-3p expression levels were markedly elevated in the model compared to control group, whereas compared to the model group, they were reduced in the EA group (**Figure 4F**).

To validate these findings, qPCR was conducted to quantify the levels of the three DE-miRNAs in peritoneal MC-EXOs in each group. The data showed that miR-149-5p, miR-22-5p and miR-1-3p were significantly upregulated in the model group compared to the control group. However, miR-149-5p and miR-22-5p were significant lower in the EA and EA+GW4869 groups than in the model group. Moreover, compared to the EA group, miR-149-5p and miR-22-5p were significantly elevated in the EA+Ucn1 and EA+C48/80 groups, but remained comparable in the EA+GW4869 group, consistent with the sequencing results (**Figure 4H**).

The target genes of the DE-miRNAs were predicted using miRwalk 3.0 (26, 27). A total of 3233 genes were detected and analyzed using the DAVID database (https://david.ncifcrf.gov/) for GO and KEGG pathway enrichment. As shown in **Figure 4G**, significant enrichment was observed in the GO category ‘bicellular tight junction’ and the KEGG pathway ‘tight junction’, indicating that the DE-miRNAs are closely associated with the regulation of tight junctions.

### 3.4 MiR-149-5p and miR-22-5p mimics cause intestinal barrier dysfunction *in vitro*

**Figure 5:**
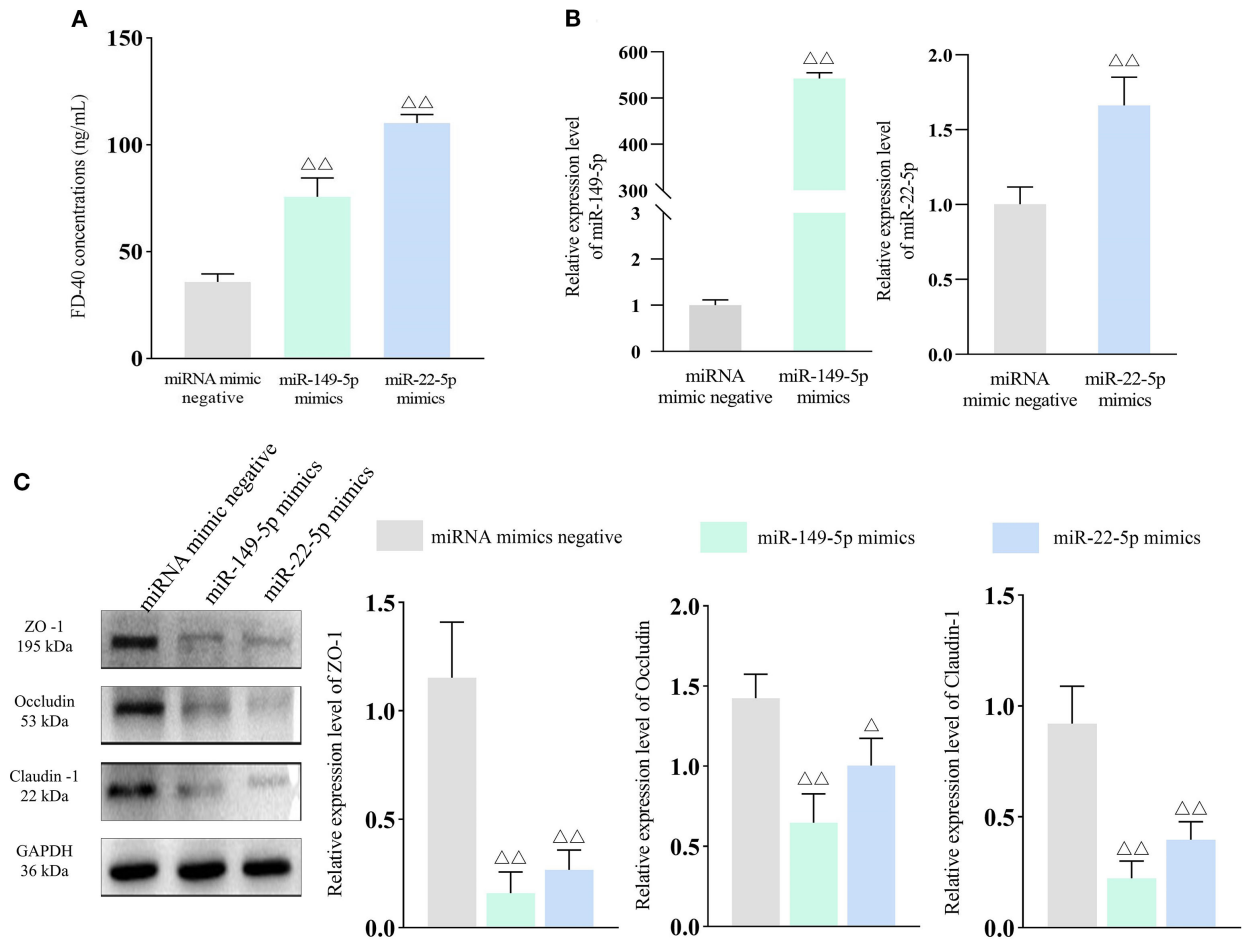
miR-149-5p and miR-22-5p expression increases intestinal permeability. Bar: mean ± SE, ^△△^ *P* < 0.01, ^△^ *P* < 0.05 vs. miRNA mimics negative group. **(A)** FD-40 concentrations in each group, n = 3; **(B)** miR-149-5p and miR-22-5p expression after transfection, n = 3; **(C)** Tight junction protein expression in each group, n = 3.

Given that miR-149-5p and miR-22-5p were the common DE-miRNAs across experimental groups, they were selected for further functional analysis. Caco-2 cells were transfected with miR-149-5p and miR-22-5p mimics to examine the effects of these miRNAs on intestinal permeability. qPCR confirmed significantly elevated expression levels of both miRNAs in transfected cells compared to the respective negative controls (*P* < 0.01), indicating successful transfection (**Figure 5B**). In addition, FD-40 concentrations were markedly higher in the miR-149-5p and miR-22-5p groups than in the miRNA negative control groups (both *P* < 0.01), demonstrating that overexpression of these miRNAs increased intestinal permeability (**Figure 5A**).

To validate the effects of miR-149-5p and miR-22-5p on tight junction proteins, WB was performed. As shown in **Figure 5C**, ZO-1, Occludin and Claudin-1 expression levels were significantly downregulated in the miR-149-5p mimics and miR-22-5p mimics groups compared to the miRNA negative control groups. Collectively, these results show that elevated levels of miR-149-5p and miR-22-5p impair barrier integrity, highlighting their potential role in IBS-D pathogenesis.

### 3.5 AAV-miR-149-5p injection abolished the protective effect of EA in IBS-D rats

**Figure 6:**
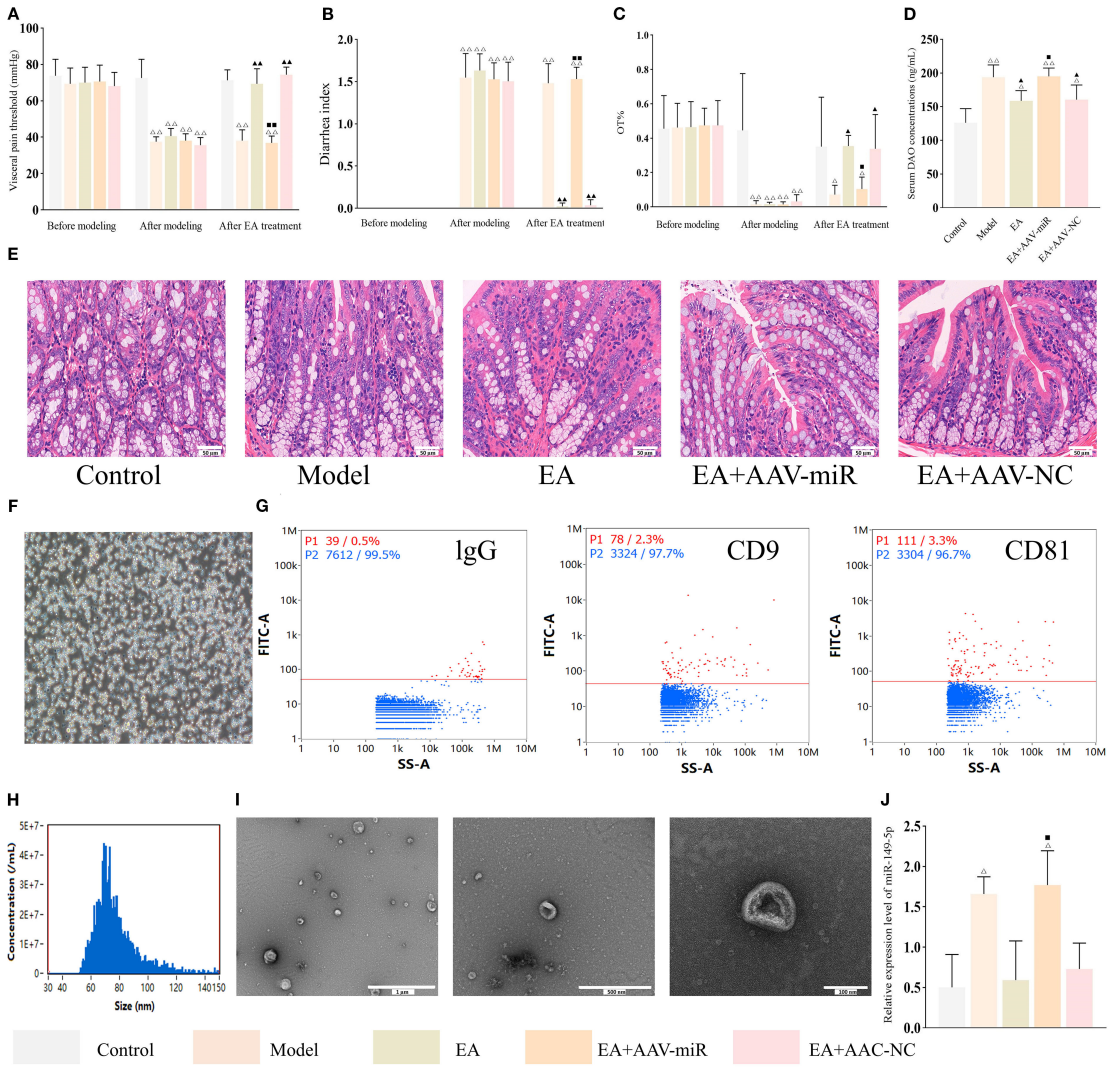
AAV-miR-149-5p injection abrogates the ameliorative effect of EA in IBS-D. Bar: mean ± SE, ^△△^ *P* < 0.01, ^△^ *P* < 0.05 vs. the control group, ^▲▲^ *P* < 0.01, ^▲^ *P* < 0.05 vs. the model group, ^◼◼^ *P* < 0.01, ^◼^ *P* < 0.05 vs. the EA group. **(A)** Visceral pain threshold, **(B)** DI, and **(C)** OT% in each group at three time points, n = 8; **(D)** Serum DAO concentrations in each group, n = 6; **(E)** Histology images of colon tissues in each group (400×), scale bar = 50 µm; **(F)** Isolation and identification of mast cells: mast cells stained strongly positive with toluidine blue and situation of mast cells culture (×100), scale bar = 100 µm; **(G)** Flow plots of IgG (negative control), CD9 and CD81 expression on MC-EXOs; **(H)** Nano-flow cytometry analysis of MC-EXO particle size; **(I)** Ultrastructural of MC-EXOs under TEM, scale bar = 1 µm (left), 500 nm (center), 100 nm (right); **(J)** Relative expression of MC-EXO miR-149-5p in each group, n = 3.

A previous study reported that miR-149-5p overexpression may regulate expressions of tight junction proteins (28) and contribute to IBS-D pathogenesis (29), suggesting the role of miR-149-5p is worth further validation. To confirm this finding, adeno-associated viruses (AAVs) overexpressing miR-149-5p were constructed and administered to IBS-D rats. As shown in **Figures 6A–C**, there were no significant differences in visceral pain threshold, DI, and OT% among groups before disease induction (all *P* > 0.05). After disease induction, all experimental groups exhibited significantly reduced visceral pain thresholds and OT%, as well as increased DI, compared to the control group (all *P* < 0.01). EA treatment reversed these effects, as the EA and EA+AAV-NC groups showed a significantly higher visceral pain threshold and OT% and a lower DI compared to the model group (all *P* < 0.01). However, the EA+AAV-miR group exhibited significantly lower visceral pain threshold and OT%, and higher DI compared to the EA group (all *P* < 0.01). Furthermore, serum DAO concentrations were significantly higher in the model group compared to the control group (*P* < 0.01), lower in the EA and EA+AAV-NC groups compared to the model group (both *P* < 0.01), and higher in the EA+AAV-miR group compared to the EA group (*P* < 0.01) (**Figure 6D**). Despite these changes, no histopathological abnormalities were observed in colon tissues in any group (**Figure 6E**).

Peritoneal mast cells were isolated from each group and identified using toluidine blue staining (**Figure 6F**). Consistent with previous findings, MC-EXOs expressed CD9 and CD81 (30), exhibited a disk-shaped morphology with central concavity, had a mean diameter of 75.33 (30–150) nm, and were present at a concentration of 2.94 × 10^9^ particles/mL (**Figures 6G–I**). qPCR analysis confirmed that miR-149-5p expression in MC-EXOs was significantly higher in the model group compared to the control group (*P* < 0.01), markedly lower in the EA and EA+AAV-NC groups relative to the model group (*P* < 0.01), and significantly increased in the EA+AAV-miR group compared to the EA group (*P* < 0.01) (**Figure 6J**).

**Figure 7:**
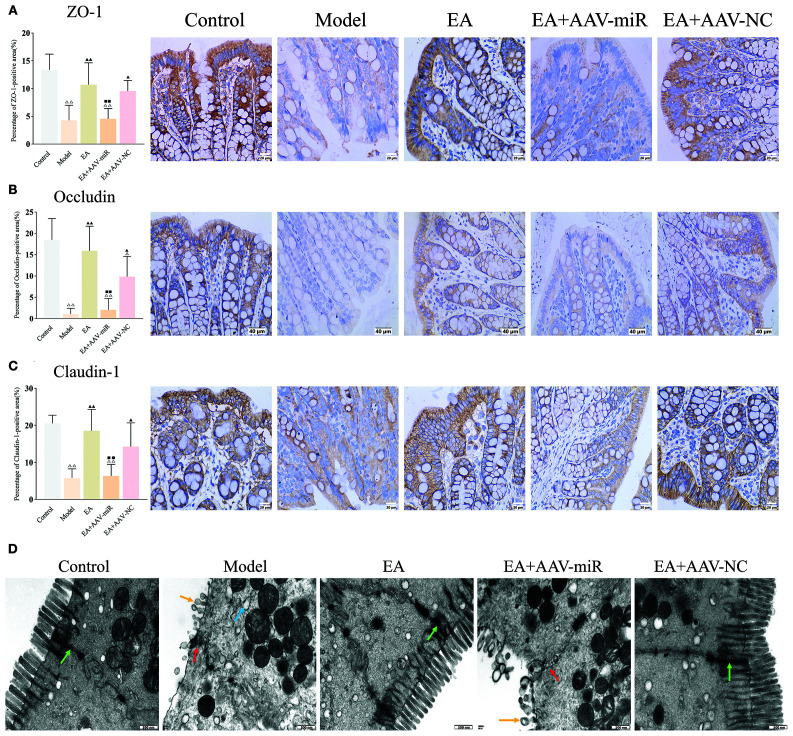
AAV-miR-149-5p injection drives intestinal barrier dysfunction. Bar: mean ± SE, ^△△^ *P* < 0.01, ^△^ *P* < 0.05 vs. the control group, ^▲▲^ *P* < 0.01, ^▲^ *P* < 0.05 vs. the model group, ^◼◼^ *P* < 0.01 vs. the EA group. **(A)** ZO-1, **(B)** Occludin, and **(C)** Claudin expression in each group, n = 6; **(D)** Ultrastructural of tight junctions under TEM (12,000×), green arrow: normal tight junctions, red arrow: impaired tight junctions, yellow arrow: destroyed microvillus, blue arrow: expanded rough endoplasmic reticulum. Scale bar = 500 nm.

IHC staining for ZO-1, Occludin and Claudin-1 demonstrated that the percentage of tight junction protein-positive areas was significantly reduced in the model group compared to the control, EA, and EA+AAV-NC groups (all *P* < 0.01), and lower in the EA+AAV-miR group compared to the EA group (*P* < 0.01) (**Figures 7A–C**). These findings were corroborated by TEM, which revealed intact tight junctions in the control, EA, and EA+AAV-NC groups, but notable disruption of tight junctions and microvilli in both the model and EA+AAV-miR groups (**Figure 7D**).

## 4 Discussion

**Figure 8:**
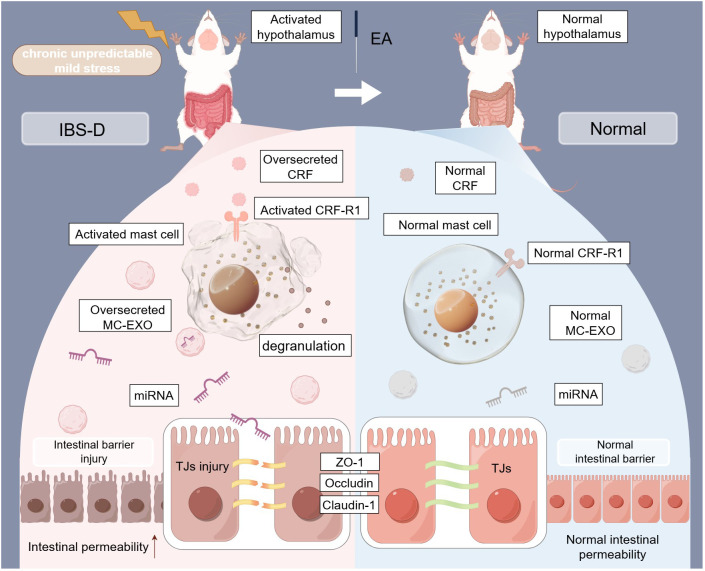
Mechanisms of EA-mediated intestinal barrier repair via the regulation of tight junctions through MC-EXO miRNAs in IBS-D rats. TJs, tight junctions; MC-EXOs, mast cell-derived exosomes; IBS-D, diarrhea-predominant irritable bowel syndrome; EA, electroacupuncture; miRNA, microRNA (This figure was created by www.figdraw.com).

The present study demonstrated that miR-149-5p carried by MC-EXOs modulates the ameliorative effect of EA on intestinal barrier function in IBS-D (**Figure 8**). As the first line of defense in the gastrointestinal tract, the intestinal mucosal barrier has garnered increased attention in IBS-D research (31, 32). Previous studies have shown that psychological stress significantly impacts intestinal barrier integrity and plays a crucial role in IBS-D pathogenesis (33, 34). Earlier work from this laboratory demonstrated that chronic stress induced diarrhea and comprised intestinal barrier function in rats, forming the basis for the IBS-D rat model using CUMS and senna solution (35). This model could mimic the symptoms of IBS-D subtypes but was limited in mimicking symptoms associated with post-infectious conditions or brain-gut axis dysfunction.

Consistent with previous findings, this study showed that EA treatment at the ST36 (*Zusanli*), ST25 (*Tianshu*), and LR3 (*Taichong*) acupoints, using a frequency of 2 Hz/15 Hz and a intensity of 0.5 mA, markedly alleviated IBS-D symptoms, including visceral hypersensitivity, diarrhea and anxiety-like behavior (35). According to previous studies, different EA protocols could have varying effectiveness (36). In the future, future investigations into sustained effects could be applied and dose-response experiments could be further employed to measure effects of varying frequency and intensity on tight junction proteins and intestinal barrier.

Mast cell degranulation is the primary mode of mast cell activation and plays a critical role in regulating intestinal barrier function (37). Tryptase is secreted during mast cell degranulation and has been shown to negatively affect intestinal barrier integrity in IBS-D rats (16, 38). Hyperactivation of CRF and CRF-R1 has also been implicated in both emotional and gastrointestinal dysfunctions (39, 40). The research team has previously found that EA inhibits mast cell activation and CRF-R1 expression and upregulate the expressions of tight junction proteins, thereby reducing intestinal permeability and promoting barrier repair (17, 24). To further explore this mechanism, CRF and mast cell agonists were used in this study. The results showed that EA effectively downregulate CRF and CRF-R1 expression, restored mast cell ultrastructure, alleviated IBS-D symptoms, and repaired the intestinal barrier. However, these therapeutic effects were significantly attenuated when CRF or mast cell agonists were administered, suggesting that the benefits of EA are mediated through inhibition of CRF/CRF-R1 and mast cell activation.

Exosomes, one of the most representative products of mast cell degranulation (41), have been shown to be a major driver of IBS pathogenesis (42). Interestingly, the present study found that the effects produced after administration of an exosome antagonist were similar to those observed in the EA-treated group, suggesting that inhibiting exosome release may replicate the therapeutic effects of EA. Research has demonstrated that exosomes derived from patients with IBS can increase cellular permeability in human colonic epithelial cells, highlighting their involvement in the regulation of intestinal barrier function (43, 44). Moreover, exosomes have been proposed as potential carriers for transmitting acupuncture signals (45). Notably, MC-EXOs have been implicated in neuroimmune regulation and are considered key mediators in the therapeutic effects of acupuncture (46). These findings further supported the critical role of MC-EXOs in EA treatment of IBS.

Numerous studies have shown that miRNAs regulate intestinal mucosal barrier function in IBS by modulating the expression of tight junction proteins (47). Notably, miRNAs are key bioactive molecules carried by MC-EXOs (48), where they are more stable than in other forms. Increasing evidence suggests that these miRNAs are involved in the regulation of intestinal barrier function (49) and may contribute to tissue injury (50). For example, mast cell-derived miR-223 downregulates CLDN8 expression in intestinal epithelial cells, thereby impairing barrier function (23). Taken together, these data indicate that MC-EXO miRNAs contribute to intestinal barrier dysfunction in IBS-D.

Both high-throughput sequencing and qPCR analyses of MC-EXOs revealed that the expression levels of miR-22-5p and miR-149-5p were significantly elevated in the model group but reduced in the EA group. These miRNAs were enriched in pathways related to tight junctions, aligning with the present findings. miR-22-5p is known to be a regulator of myocardial, pulmonary, and hepatic functions (51–53). miR-1-3p has been extensively studied in cancer, particular gastric cancer (54, 55), and has been linked to intestinal dysfunction in the aging colon (56). On the other hand, miR-149-5p has been implicated in both cancer biology and intestinal barrier function (57). Also, according to the results of PCR validation, miR-1-3p showed no significant change among three groups, thus it is excluded in further validation in this study.

To further validate the sequencing results, an *in vitro* model of the intestinal epithelial barrier was established using the Caco-2 cell line. It was found that cells transfected with miR-149-5p and miR-22-5p mimics exhibited decreased expression of tight junction proteins and increased cellular permeability. miR-149-5p has been shown to be involved in various physiological processes, including cell proliferation and inflammatory responses (58, 59). These is evidence suggested that miR-149-5p played a key role in regulating expressions of tight junction proteins (28). More importantly, miR-149-5p is significantly upregulated in the serum of patients with IBS-D and Its level is highly likely to be regulated by EA treatment (29). To explore the role of miR-149-5p *in vivo*, AAV-miR-149-5p was constructed and administered to IBD-S rats. The results showed that AAV-miR-149-5p injection abolished the protective effect of EA on the intestinal barrier, further supporting the deleterious role of MC-EXO miR-149-5p in EA-mediated restoration of intestinal barrier integrity.

Previous studies have reported that miR-149-5p is significantly upregulated in the IECs of septic rats, resulting in inflammation, tissue injury, and intestinal barrier disruption (60). Additionally, miR-149-5p injection in MCAO rats significantly altered the expression of ZO-1 and Occludin (28), suggesting that miR-149-5p impairs barrier integrity by downregulating these tight junction proteins. Consistent with these results, the present findings further elucidated the role of MC-EXO miR-149-5p in intestinal dysfunction associated with IBS-D and highlighted the barrier-protective effect of EA.

Pathway analysis revealed that predicted targets of differentially expressed miRNAs are enriched in “Regulation of Actin Cytoskeleton”, a pathway directly affecting tight junction assembly (61). Patients with IBS-D display decreased levels of cytoskeletal components in their colons (62). Recent studies indicate that MCs could play a crucial role in regulating the cytoskeleton (63). Accumulating evidences have suggested that EA might play a positive role in regulating cytoskeleton (64). Moreover, it is reported that miR-149-5p might serve a key role in regulating cytoskeleton (65). Therefore, these will also be the focus of our next research.

Current major IBS-D treatments including 5-HT_3_ antagonists and probiotics have obvious limitations. It is reported 5-HT_3_ antagonists could increase the risk of ischemic colitis by causing barrier injury (66). Also, although probiotics generally support barrier function, specific strains may increase paracellular permeability (67). Our findings demonstrate that overexpression of exosomal miR-149-5p impairs intestinal barrier function in IBS-D by upregulating key tight junction proteins. This endogenous mechanism offers distinct advantages without neurological side effects, potentially synergizing with existing therapies by correcting their barrier-damaging side effects, and demonstrating physiological relevance evidenced by its natural upregulation in the pathogenesis of human IBS-D.

Several limitations must be noted in the present study. First, although *in vitro* and *in vivo* experiments using agonists, antagonists, and AAV were performed to demonstrate the essential role of MC-EXO miRNAs, future studies in Rab27a gene knockout rats, luciferase reporter assays and AGO2-RIP qPCR are warranted to validate the screening results, and MC-EXO miR-149-5p levels in human IBS-D colonic mucosa or serum can be detected to strengthen the reliability of the findings. Second, while both miR-149-5p and miR-22-5p were identified and validated *in vitro*, only miR-149-5p was further investigated using AAV. Therefore, future work should focus on elucidating the role of miR-22-5p. Also, FACS-sort mast cell-specific markers or the use of mast cell-specific Cre-lox tracing to label exosomes could be applied to exclude non-mast cell-derived exosomes. Furthermore, off-target effects of EA on non-dysregulated miRNAs still remains unexplored, this must be addressed in future study. Last, given the importance of mast cells and their crosstalk with other immune cells in the pathogenesis of IBS-D (68, 69), subsequent studies are needed to explore these interactions in greater detail.

## 5 Conclusion

MC-EXO miR-149-5p might modulate the restorative effect of EA on intestinal barrier function by downregulating the expression of tight junction proteins, serving as a potential therapeutic target for IBS-D.

## Data Availability

The data presented in the study are deposited in the CNSA repository, accession number CNP0008009. The data can be found here: https://db.cngb.org/data_resources/project/CNP0008009/
